# Young Epidemiologists’ Attitude towards Personal Data Protection

**DOI:** 10.2188/jea.16.90

**Published:** 2006-03-14

Dear Sir:

The Personal Information Protection Act took effect on April 1, 2005. With increasing emphasis on the protection of personal data, epidemiologists are now expected not only to ensure scientific validity and ethical acceptability of research but also to acquire technical knowledge and skills required to ensure the protection of personal data obtained in the course of research. In view of this fact, a questionnaire survey was conducted among members of the Young Epidemiologists Society for Discussing the Future of Epidemiology (YES), a subdivision of the Japanese Epidemiological Association (JEA), members of which may be very interested in the future direction of epidemiology,^1^ with a view to identifying the epidemiologists’ need for (1) professional training in personal data protection and (2) a scheme for accrediting data protection officers (DPOs).

In a YES meeting held on January 20, 2005, a survey was conducted using a self-administered questionnaire to examine the YES members’ attitude towards personal data protection. The questionnaire included questions about the following items: (1) who should assume the responsibility for the protection of personal data collected from epidemiologic research, (2) information that requires protection by DPOs, (3) skills, knowledge, and personality required of DPOs in epidemiologic research and the need for a DPO accreditation scheme, (4) penalties for the leakage of personal information, and (5) whether or not JEA should provide training programs for DPOs or establish a DPO accreditation scheme. Answers obtained for open-ended questions were analyzed and examined using the KJ-method.

Although YES was originally meant to be a society of young researchers, there is no age limit for participants. In fact, YES consists of 151 members whose ages range from 20s to 50s years. Of the 59 members participated in the meeting held in 2005, 49 (87%) provided valid responses. Twenty-eight respondents were male and 23 respondents were in their 30s. Thirty respondents had been involved in epidemiologic research for 0-5 years, seven respondents for 6-10 years, and six respondents for 11-15 years. Thirty-seven respondents belonged to educational/research institutions.

Thirty-five respondents (71%) replied that principal investigators should be responsible for the protection of personal data, while nine (18%) replied that DPOs should be responsible for data protection. When asked who should assume the role of DPO, 11 respondents (22%) suggested that PIs should assume the role of DPOs, while eight (16%) suggested that someone from outside the research team not belonging to the same research circle should act as DPOs. Some even suggested that DPOs should be invited from a non-affiliated third-party institution.

Forty-four respondents (90%) replied that genetic information requires protection by DPOs, 29 (59%) replied that clinical data should be protected, 28 (57%) replied that patient financial data (including income data) should be protected by DPOs (Table 1).

**Table 1.** Information that requires protection by data protection officers (n=49).

	number (%)
Genetic information	44 (90)
Clinical data	29 (59)
Patient financial data (including income data)	28 (57)
Addresses/phone numbers	26 (53)
Human specimen collected for purposes other than genetic research	24 (49)
Responses obtained from questionnaire surveys	16 (33)

Forty-six respondents (94%) indicated that DPOs should be familiar with various laws and regulations related to the protection of personal data (i.e., Personal Information Protection Act), 40 (82%) indicated that DPOs should have adequate understanding of data protection requirements and should be able to implement adequate data protection measures (Table 2).

**Table 2.** Skills, knowledge, and personality required of data protection officers in epidemiological research (n=49).

	number (%)
Understanding of various laws and regulations related to the protection of personal data	46 (94)
Adequate understanding of data protection requirements and ability to implement adequate data protection measures	40 (82)
Adequate understanding and knowledge of epidemiologic research	32 (65)
Responses obstrong sense of responsibility	26 (53)
Good personality	21 (43)
Others*	2 (4)
Multiple responses allowed	

* Others (comments): Ethical values (one respondent) or personality/sense of responsibility (one respondent) does not influence one’s aptitude for the role of data protection officers.

Thirty-one respondents (63%) indicated that there should be a DPO accreditation scheme for the following two reasons: (1) Epidemiologists and DPOs should have certain levels of understanding regarding the protection of personal data (nine respondents), and (2) confidentiality of individuals participating in epidemiologic research should be given adequate protection (four respondents). On the other hand, 18 respondents (37%) indicated that there was no need for a DPO accreditation scheme for the following two reasons: (1) adequate protection can be given to personal information by implementing strict rules of confidentiality, by providing adequate trainings and by imposing penalties to deter noncompliance (three respondents), and (2) DPOs are not legal professionals, and thus they do not have to be experts on Personal Information Protection Act (two respondents). Of the 31 respondents who indicated that there should be a DPO accreditation scheme, 23 (74%) indicated that the scheme should be provided by JEA, while 14 (45%) indicated that it should be provided by the national government.

When asked about qualifications required for accreditation as DPOs, 24 respondents (52%) indicated that DPOs should be appropriately trained, while 18 (39%) indicated that one should pass a test in order to qualify as a DPO. The following types of training programs were suggested by the respondents: (1) Training sessions + a test (seven respondents), (2) JEA-led training sessions (two respondents), etc.

Following punishment was suggested for DPOs who have failed to prevent information leakage. In this respect, 20 respondents (41%) suggested legal punishment, 15 respondents (31%) suggested issuance of warning notices by JEA, 11 respondents (22%) suggested prohibiting the DPO from participating in research activities, and nine respondents (18%) suggested deprivation of authorship. However, two respondents indicated that such penalties were too harsh.

Through a series of discussions, surveys and round-table meetings that have been held among epidemiologists in Japan since the late 1990s, it has become clear that young epidemiologists in Japan recognize the importance of enhancing public awareness and support of epidemiologic research, and believe that the goal of epidemiologic research should be to improve and promote public health.^1^ In the past, due to the nature of epidemiologic research, little ethical or social issues were expected to arise from the conduct of epidemiologic studies. However, from the beginning of the late 1990s, epidemiologic studies began to involve the use of various sensitive data, including genetic information. Afterwards, informed consent began to assume a great importance in epidemiologic research. In 2002, a group of epidemiologists published ethics guidelines.^2^ In June, 2002, the Ministry of Education, Culture, Sports, Science and Technology and the Ministry of Health, Labour and Welfare collaborated to establish the *Guidelines* on *Ethics for Epidemiologic Research*. Consequently, an ethics committee has come to play a critical role in epidemiologic research. The Personal Information Protection Act, which is designed to prevent leakage of personal data, took effect in April 2005. Although methods of data protection have long been left to the discretion of individuals holding the data, the time has come now for all researchers to reconsider the ways in which sensitive personal data are handled in research.^3^

However, opinion was divided over who should assume the responsibility of DPOs. Although the majority of the respondents replied that PIs should be responsible for personal data protection, some respondents claimed that someone other than principal investigators should assume the responsibility or that DPOs should be invited from a third-party institution. Further discussion is needed among PIs, researchers and an appropriate ethics committee to determine who should assume the role of DPO.

Most respondents expressed the need for a highly effective data protection system that ensures the protection of data obtained from epidemiologic research. They indicated that JEA should play a leading role in establishing such a system in Japan, ie, national level and local government level.

One should bear in mind that the results obtained from the present study do not necessarily represent the opinion of all JEA members or epidemiologists in general. However, in epidemiologic studies conducted in non-hospital settings involving a large number of individuals, special care should be exercised in the informed consent and data protection processes. In order to ensure that privacy and confidentiality of study participants are protected throughout the course of the research, DPOs should not only be adequately trained but should also have appropriate qualifications. Furthermore, the implementation of an effective data protection system may help boost the public image of epidemiology and could potentially lead to increased public support for JEA. However, there is a number of issues to be addressed before such a system could become a reality, which include (1) developing appropriate training methods as well as a system for evaluating the achievement of participants, (2) securing a sufficient number of staff to run the system, and iii) establishing penalties for information leakage. Overall, the surveyed YES members showed a high level of awareness regarding the importance of personal data protection. Epidemiologists should work together towards the early realization of an appropriate data protection system.

## Notes

Gen Kobashi (Department of Health for Senior Citizens, Hokkaido University Graduate School of Medicine, N15W7, Sapporo 060-8638, Japan. [e-mail: genkoba@med.hokudai.ac.jp]), Tsutomu Hoshuyama (Department of Environmental Epidemiology, Institute of Industrial Ecological Sciences, University of Occupational and Environmental Health), Kaori Ohta (Department of Public Health, Chiba University Graduate School of Medicine), Hiroki Sugimori (Department of Preventive Medicine, St. Marianna University School of Medicine), Izumi Oki (Department of Public Health, Jichi Medical School), Hideyuki Kanda (Department of Hygiene & Preventive Medicine, Fukushima Medical University), Mariko Naito (Department of Preventive Medicine/Biostatistics and Medical Decision Making, Nagoya University Graduate School of Medicine), Soshi Takao (Department of Hygiene and Preventive Medicine Okayama University Graduate School of Medicine), and Akiko Tamakoshi (Department of Preventive Medicine/Biostatistics and Medical Decision Making, Nagoya University Graduate School of Medicine).
